# Targeting Paraprotein Biosynthesis for Non-Invasive Characterization of Myeloma Biology

**DOI:** 10.1371/journal.pone.0084840

**Published:** 2013-12-23

**Authors:** Katharina Lückerath, Constantin Lapa, Annika Spahmann, Gerhard Jörg, Samuel Samnick, Andreas Rosenwald, Herrmann Einsele, Stefan Knop, Andreas K. Buck

**Affiliations:** 1 University Wuerzburg, Medical Center, Department of Nuclear Medicine, Wuerzburg, Germany; 2 University Wuerzburg, Medical Center, Department of Hematology and Oncology, Wuerzburg, Germany; 3 University Wuerzburg, Institut of Pathology, Wuerzburg, Germany; Karolinska Institutet, Sweden

## Abstract

**Purpose:**

Multiple myeloma is a hematologic malignancy originating from clonal plasma cells. Despite effective therapies, outcomes are highly variable suggesting marked disease heterogeneity. The role of functional imaging for therapeutic management of myeloma, such as positron emission tomography with 2-deoxy-2-[^18^F]fluoro-D-glucose (^18^F-FDG-PET), remains to be determined. Although some studies already suggested a prognostic value of ^18^F-FDG-PET, more specific tracers addressing hallmarks of myeloma biology, e.g. paraprotein biosynthesis, are needed. This study evaluated the amino acid tracers *L*-methyl-[^11^C]-methionine (^11^C-MET) and [^18^F]-fluoroethyl-*L-tyrosine* (^18^F-Fet) for their potential to image myeloma and to characterize tumor heterogeneity.

**Experimental Design:**

To study the utility of ^11^C-MET, ^18^F-Fet and ^18^F-FDG for myeloma imaging, time activity curves were compared in various human myeloma cell lines (INA-6, MM1.S, OPM-2) and correlated to cell-biological characteristics, such as marker gene expression and immunoglobulin levels. Likewise, patient-derived CD138^+^ plasma cells were characterized regarding uptake and biomedical features.

**Results:**

Using myeloma cell lines and patient-derived CD138^+^ plasma cells, we found that the relative uptake of ^11^C-MET exceeds that of ^18^F-FDG 1.5- to 5-fold and that of ^18^F-Fet 7- to 20-fold. Importantly, ^11^C-MET uptake significantly differed between cell types associated with worse prognosis (e.g. t(4;14) in OPM-2 cells) and indolent ones and correlated with intracellular immunoglobulin light chain and cell surface CD138 and CXCR4 levels. Direct comparison of radiotracer uptake in primary samples further validated the superiority of ^11^C-MET.

**Conclusion:**

These data suggest that ^11^C-MET might be a versatile biomarker for myeloma superior to routine functional imaging with ^18^F-FDG regarding diagnosis, risk stratification, prognosis and discrimination of tumor subtypes.

## Introduction

Multiple myeloma (MM), classified as a post-germinal center Non-Hodgkin`s lymphoma, is a hematological neoplasm originating from plasma cells. MM accounts for approximately 1% of all cancers and around 10% of hematological malignancies [1,2]. Despite recent advent of new therapeutics enabling more durable partial or complete remissions, almost all patients eventually relapse and die from their disease. A critical question remains whether - not yet clearly defined - subgroups of patients can benefit from more aggressive therapies. Due to high inter- and intra-patient tumor heterogeneity, identification of molecular lesions driving myeloma in individual patients is essential for the development of novel therapeutic algorithms [3-5]. Besides planar x-ray, the role of imaging for therapeutic management of MM and risk stratification remains to be determined. Several studies have demonstrated the usefulness of positron emission tomography (PET) using the radiolabeled glucose analog 2-deoxy-2-[^18^F]fluoro-D-glucose (^18^F-FDG) for diagnosis, staging and prognostication, leading to implementation into the revised Salmon/Durie staging system (Salmon/Durie PLUS) [6-10]. 

However, ^18^F-FDG PET has limited sensitivity and specificity: glucose uptake in inflammatory lesions can lead to false positive findings; the generally low metabolic activity of MM might account for false negative results, especially in case of diffuse bone marrow involvement [11]. MM is characterized by excess production of aberrant immunoglobulins (M-protein). Therefore, radiotracers addressing paraprotein biosynthesis and/or amino acid transport might serve as surrogate markers reflecting metabolic activity of the disease and, hence, prove useful for assessing response to therapy and prognosis in individual patients.

This study aimed at evaluating the amino acid tracers *L*-methyl-[^11^C]-methionine (^11^C-MET) and [^18^F]-fluoroethyl-*L-tyrosine* (^18^F-FET) for their potential to characterize MM lesions non-invasively. Time activity curves of ^11^C-MET, ^18^F-FET and ^18^F-FDG were compared in various human myeloma cell lines and correlated to hallmarks of MM biology, including levels of immunoglobulin (Ig) light chains, proliferation rate, as well as CD138 and CXCR4 expression. In a more physiological model, primary CD138^+^-plasma cells were analyzed regarding retention of imaging biomarkers. Uptake patterns were correlated to biomedical features of individual patient samples. Our data suggest that ^11^C-MET represents a versatile imaging biomarker for MM with the potential to specifically detect MM lesions using PET and to discriminate tumor subtypes. 

## Materials and Methods

### Ethics statement

All experiments involving human material were approved by the ethics committee of the University Wuerzburg (#192/12). Bone marrow biopsies from patients diagnosed with MM were taken after obtaining informed written consent from each patient.

### Cell culture

The human myeloma cell line INA-6 [12] was a gift from the Dept. of Hematology, University Hospital Wuerzburg. OPM-2 (DSMZ no. ACC50) cells were purchased from the German Collection of Microorganisms and Cell Culture (DSMZ, Braunschweig, Germany) and MM.1S (ATCC no. CRL-2974) were obtained from LGC Standards (Wesel, Germany). Cell lines were cultured in Roswell Park Memorial Institute Medium 1640 (supplemented with 10% FCS, 2mM L-glutamine, 1mM sodium pyruvate, 100 U/mL penicilline and 100 µg/mL streptomycine; all media and supplements: Invitrogen, Darmstadt, Germany) at 37 °C in a 5% CO_2_, humidified atmosphere. Additionally, 2.7 ng/mL hrIL-6 (Miltenyi, Bergisch-Gladbach, Germany) were added to cultures of INA-6 cells. Cell line identity was confirmed at the DSMZ (July 2013) by testing for the expression of eight different short tandem repeat loci according to the guidelines for authentication of human cell lines and, additionally, by examining for presence of rodent mitochondrial DNA sequences. Regular testing of cell cultures using the Venor GeM Mycoplasma Detection Kit (Sigma-Aldrich, Taufkirchen, Germany) ensured absence of contamination with mycoplasma. 

### Isolation of CD138^+^-plasma cells

CD138^+^-plasma cells were isolated from bone marrow aspirates of 19 patients diagnosed with MM by Ficoll density gradient centrifugation (density 1.007; Sigma-Aldrich, Taufkirchen, Germany) and positive selection using CD138^+^-micro beads and MACS technology (Miltenyi, Bergisch-Gladbach, Germany) after obtaining informed written consent. Purity of isolated cells was controlled by flow cytometry using an anti-hCD138^+^-APC antibody (Miltenyi, Bergisch-Gladbach, Germany). Isolated cells were diluted in PBS to a defined concentration and directly analyzed in uptake experiments.

### Flow cytometric analyses

Single cell suspensions were stained with fluorochrome conjugated antibodies against hCD138^+^-APC (Syndecan; clone B-B4) or hCXCR4-PE (hCD184; clone 12G5; Miltenyi, Bergisch-Gladbach, Germany) and analyzed with a BD FACSCalibur flow cytometer using the BD CellQuest software (Beckton Dickinson, Heidelberg, Germany). Intracellular staining of immunoglobulin kappa and lambda light chains was performed using anti-hIg kappa light chain-APC (clone IS11-24D5) and anti-hIg lambda light chain-FITC (clone IS7-24C7) antibodies with the Inside Stain Kit from Miltenyi (Bergisch-Gladbach, Germany) according to the manufacturer's instructions.

### Cell proliferation assay

Cells were seeded at a density of 1*10^5^ cells per well in a 96-well plate in triplicates, grown for 48 h and were subsequently fixed with 70% ethanol. After overnight storage at 4 °C, cells were washed and stained with rabbit-anti-hKi67-FITC antibody (clone SP6; abcam, Camebridge, UK) according to the manufacturer's instructions. Geometric mean fluorescent activity (GeoMean) of samples was quantified with a BD FACSCalibur flow cytometer using the BD CellQuest software (Beckton Dickinson, Heidelberg, Germany) and corrected for background staining.

### Synthesis of ^18^F-FDG, ^18^F-FET and ^11^C-MET

Radiopharmaceuticals were produced in house with a 16 MeV Cyclotron (GE PETtrace 6; GE Healthcare, Milwaukee, USA). ^18^F-FDG was synthesized using GE FASTlab methodology according to the manufacturer‘s instructions. ^18^F-FET was synthesized on a GE TRACERlab FX-FN as previously described by Bourdier et al. [13]. ^11^C-MET was synthesized on a GE TRACERlab FX-C Pro by on-column ^11^C-methylation of *L*-homocysteine with ^11^CH_3_I according to the procedures described by Kniess [14] and Gomzina and co-workers [15]. Before use, radiochemicals were analyzed by HPLC for radiochemical identity and purity.

### Cellular uptake experiments

Sub-confluent cell cultures were harvested and adjusted to a concentration of 400.000 cells/ 500 µL PBS per sample. Radioactive substances were diluted to 1*10^6^ counts per minute (cpm)/ 50 µL PBS. After addition of 1*10^6^ cpm, samples were incubated for various times up to 120 min at 37 °C. Tracer uptake was stopped by incubation on ice, followed by washing twice with PBS to remove residual radioactivity. Intracellular radioactivity was quantified using a semi-automated gamma-counter (Wallac 1480-Wizard, Perkin Elmer, Rodgau, Germany). All samples were measured in triplicates. Background activity- and decay-corrected data were expressed counts per minute (cpm) per 1000 cells.

### Statistical analysis

Statistical significance was assessed using Kruskal-Wallis-testing and posthoc analysis. A p-value of <0.05 was considered to be statistically significant. Analysis of correlation was done according to Pearson.

## Results

### Hallmarks of MM biology in myeloma cell lines

**Figure 1:**
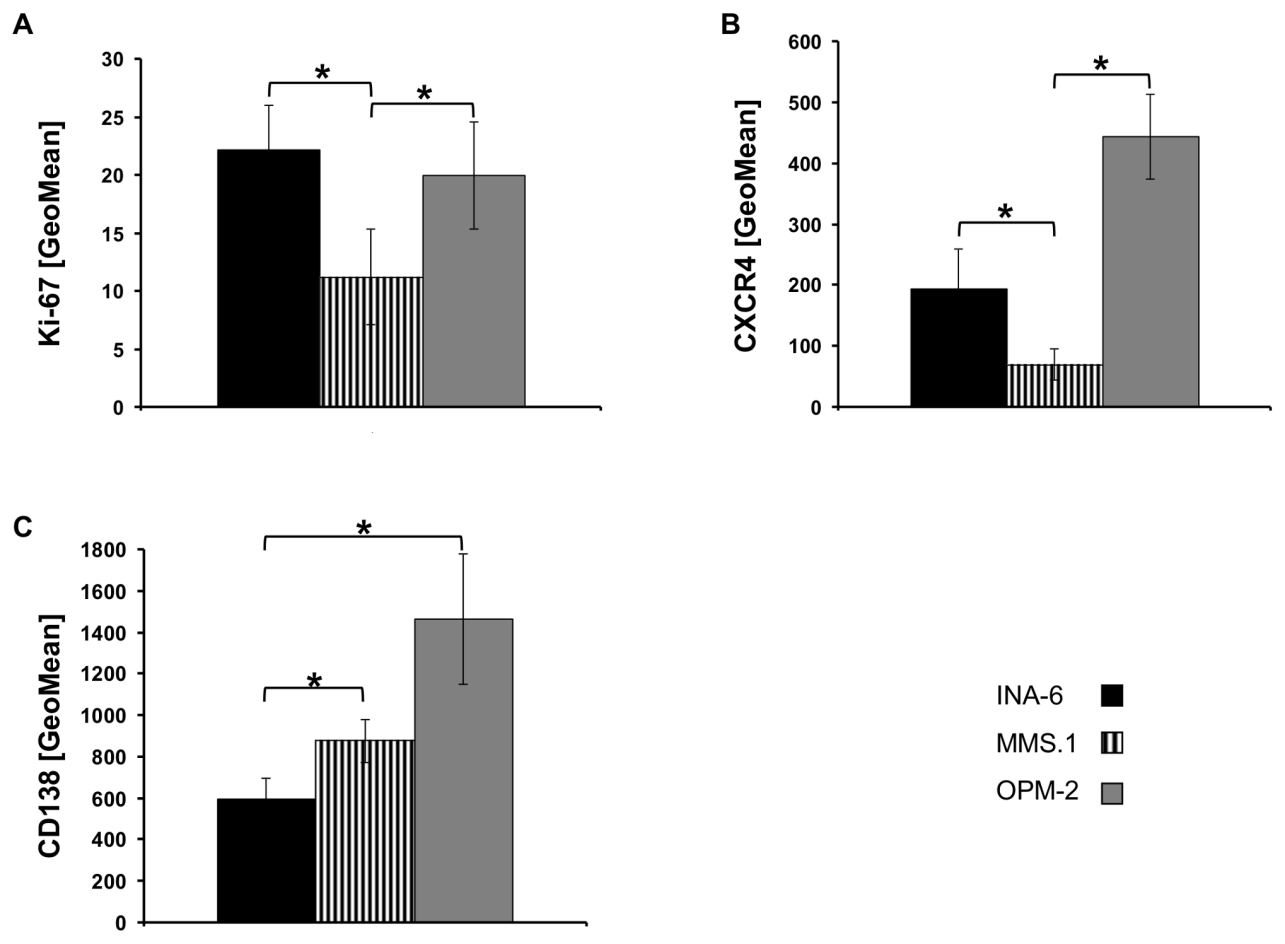
Hallmarks of MM-biology in MM-cell lines. (A) Proliferation rate. Cells were stained with anti-hKi67 FITC antibody and geometric mean fluorescent intensity (GeoMean) was quantified by FACS. All samples were analyzed in duplicates and background corrected (n=4). Cell surface expression of CXCR4 (B) and CD138^+^ (C) was analyzed by FACS. Cells were stained with an anti- hCXCR4-PE or anti- hCD138-APC antibody in duplicate, background-corrected and GeoMean was quantified (n=5). Columns represent mean values and error bars the standard deviation. Asterisk indicate statistically significant differences (p <0.05).

To reflect MM heterogeneity, MM cell lines with different clinical and cell-biological characteristics were selected (table 1). Cell lines were analyzed regarding hallmarks of MM pathology, such as proliferation rate, cell surface expression of CD138 and of CXCR4. The proliferative capacity, as assessed by flow cytometric Ki67-staining, differed significantly (p <0.05) between MM1.S *versus* OPM-2 and INA-6 cells, with the latter two growing roughly 2.5-times faster (Figure 1A). CXCR4, a homing factor for myeloma cells, was most abundant on OPM-2 cells; in contrast, INA-6 expressed only half as much CXCR4 and MM1.S cells approximately seven times less (Figure 1B). Quantification of the adhesion molecule CD138 revealed high cell surface levels on OPM-2 cells and markedly lower expression on MM1.S and INA-6 (Figure 1C).

**Table 1:** Characteristics of MM-cell lines reflect tumor heterogeneity.

**cell line**	**INA-6**	**MM1.S**	**OPM-2**
**reference**	Burger (1994)	ARCC CRL-2974	DSMZ ACC50
**species**	human	human	human
**diagnosis**	MM	MM	MM
**Ig**	IgG κ	IgA λ	IgG λ
**growth**	suspension	partially adherent	suspension
**misc.**	IL-6 dependent	dexamethasone sensitive	t(4;14) hypertriploid

### Intracellular immunoglobulin light chain levels

As MM is characterized by excess production of aberrant immunoglobulins, intracellular levels of kappa and lambda light chains were evaluated. In agreement with their origin (table 1), INA-6 cells stained positive for Ig kappa light chains, while all other cell lines produced Ig lambda light chains. Flow cytometric quantification demonstrated varying intracellular abundance of the respective light chains with increasing levels from INA-6 to MM1.S and OPM-2 cells (1 : 2 : 4; Figure 2). 

**Figure 2:**
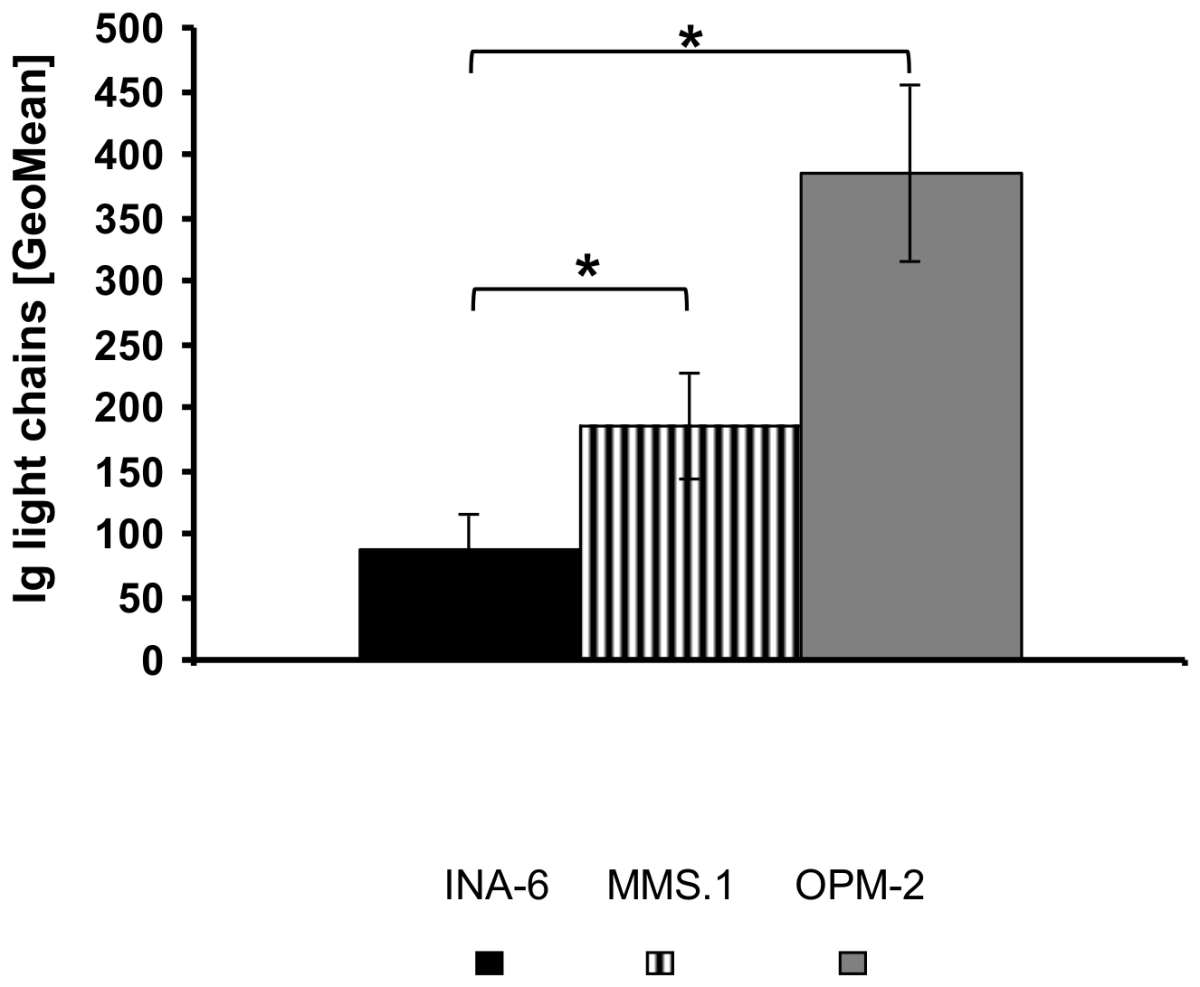
Immunoglobulin κ/λ light chain levels. Intracellular levels of either λ- (MM1.S, OPM-2) or κ- (INA-6) immunoglobulin light chains were determined by FACS analysis (GeoMean) using anti-Ig λ-FITC- and anti-Ig κ-APC antibodies. Background-corrected means ± standard deviation are shown (n=7). Asterisk indicate statistically significant differences (p <0.05).

### Uptake of ^11^C-MET and ^18^F-FET by MM cell lines in comparison to ^18^F-FDG

**Figure 3:**
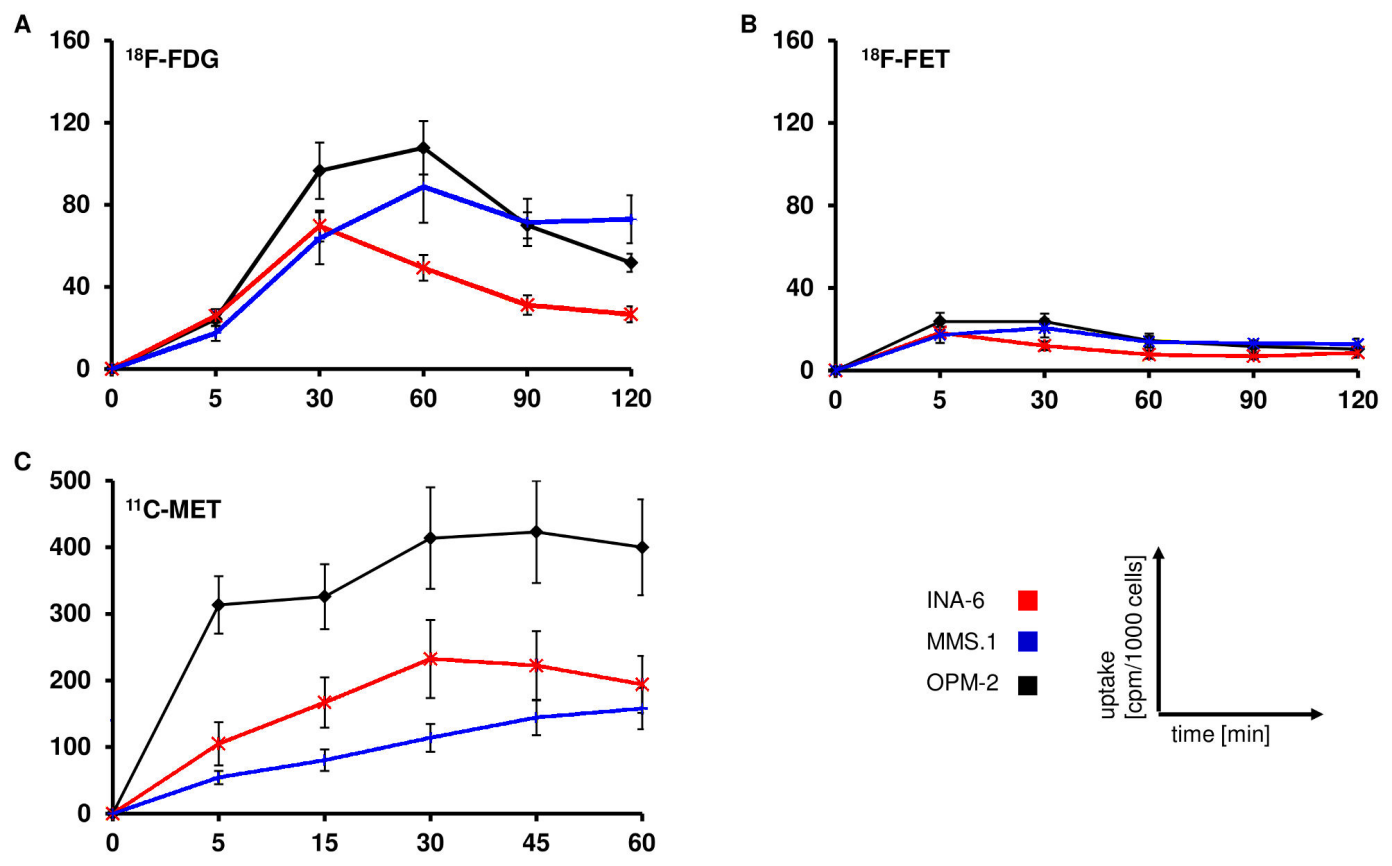
Uptake of ^11^C-MET and ^18^F-FET by MM-cell lines in comparison to ^18^F-FDG. Intracellular radioactivity following incubation with ^18^F-FDG (A), ^18^F-FET (B) or ^11^C-MET (C) was quantified using a gamma-counter. Relative uptake of background- and decay-corrected triplicate-samples was expressed as cpm per 1000 cells (mean ± sem; n=5).

^18^F-FDG-PET is of value for the detection of MM-lesions, but radiotracers addressing the characteristic paraprotein biosynthesis might be more appropriate to reflect metabolic activity of the disease. Maximum uptake of ^18^F-FDG approximated 70-100 cpm/1000 cells in all cell lines and was reached after 30 min (INA-6) or 60 min (OPM-2, MM.1S), respectively. Thereafter, slightly decreasing radiotracer retention was observed (Figure 3A). 

Levels of intracellular ^18^F-FET were significantly lower than those of ^18^F-FDG, with a maximum level of ~20 cpm/1000 cells (Figure 3B). Efflux of ^18^F-FET occurred rapidly. The highest retention was observed for ^11^C-MET and ranged between 144 cpm/1000cells for MM1.S cells (45 min), 232 cpm/1000cells for INA-6 (30 min) and 422 cpm/1000cells for OPM-2 cells (45 min). Already after 5 minutes post tracer application, relative uptake of ^11^C-MET exceeded maximal ^18^F-FDG retention drastically. Interestingly, ^11^C-MET levels discriminated two groups: methionine-uptake by OPM-2 cells was significantly higher than by INA-6 and MM.1S cells (Figure 3C). 

### Validation of ^11^C-MET, ^18^F-FET and ^18^F-FDG as surrogate markers of MM biology in CD138^+^-plasma cells

**Figure 4:**
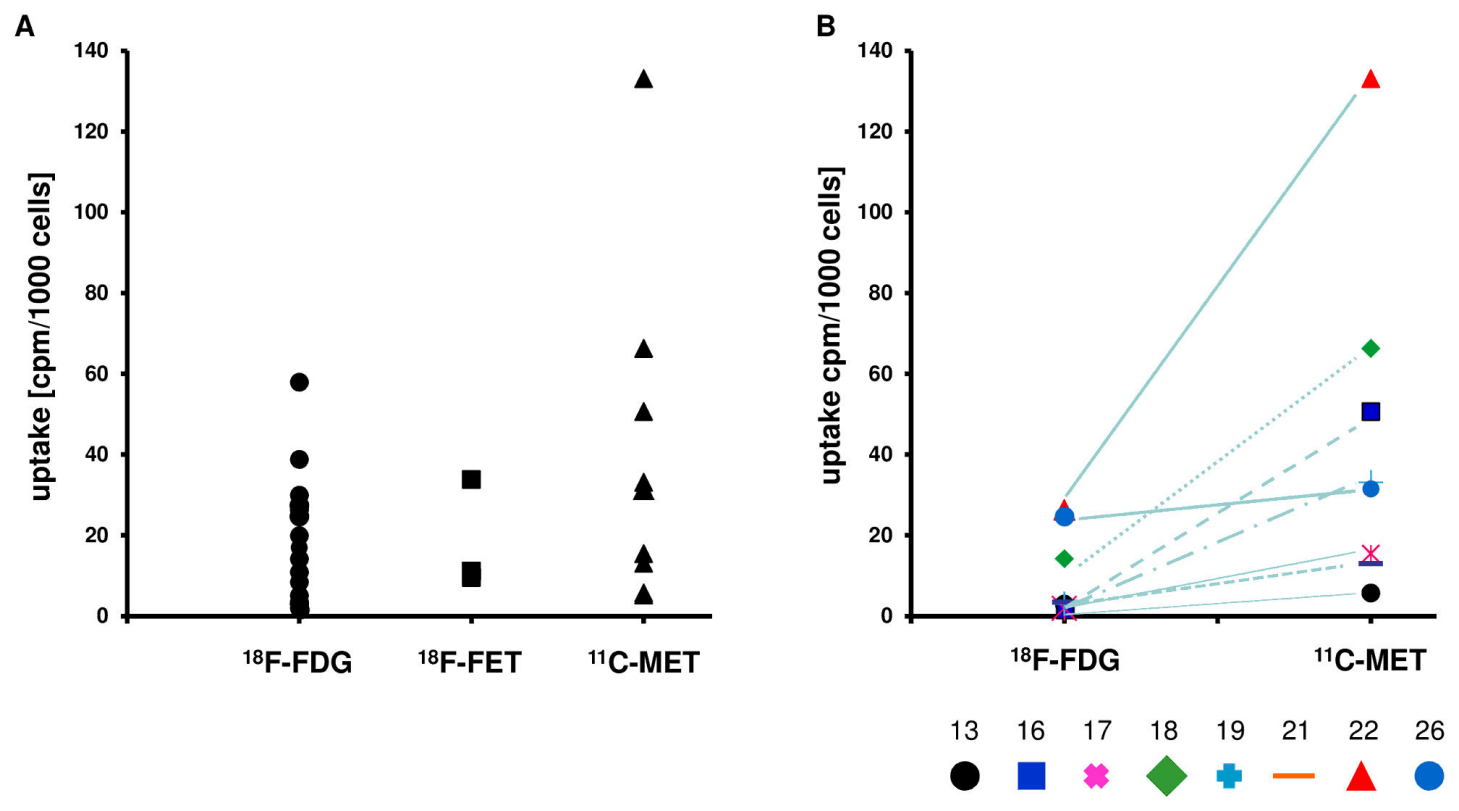
^11^C-MET is superior to ^18^F-FET and ^18^F-FDG in CD138^+^-plasma cells. CD138^+^-plasma cells were incubated with either ^18^F-FDG, ^18^F-FET or ^11^C-MET for 60 min and intracellular radioactivity was quantified using a gamma-counter. Relative uptake of background- and decay-corrected samples was expressed as cpm per 1000 cells. Whenever possible, bone marrow samples were split and one half of the sample was incubated with ^18^F-FDG, the other with either ^18^F-FET (patients no 7, 10, 11) or ^11^C-MET (patients no. 13, 16, 17, 18, 19, 21, 22, 26). (A) ^18^F-FDG, ^18^F-FET and ^11^C-MET uptake by CD138^+^ PCs. Data from all samples analyzed are shown. (B) Direct comparison of ^18^F-FDG and ^11^C-MET uptake in split samples. Lines indicate corresponding samples from one patient.

Next we set out to validate our findings using patient-derived MM cells (table 2). The strongly limited cell number in most samples only permitted single time point analyses. Whenever cell number allowed, cells isolated from one patient were split and one half was incubated for 60 min with either ^11^C-MET (patients no. 13, 16, 17, 18, 19, 21, 22, 26) or ^18^F-FET (patients no 7, 10, 11), whereas the second half was incubated with ^18^F-FDG for direct comparison between test and standard tracer. In agreement with the results in established cell lines, the amount of ^18^F-FET retained by primary MM-cells after 60 min tended to be less than that of ^18^F-FDG (Figure 4A). However, direct intra-sample comparison did not reveal clear differences between ^18^F-FET- and ^18^F-FDG-retention. Contrarily, primary MM cells had a markedly enhanced capacity to take up ^11^C-MET (Figure 4A). This latter finding was especially intriguing when directly comparing ^18^F-FDG and ^11^C-MET data (Figure 4B). Furthermore, higher ^11^C-MET retention in a sample tended to be accompanied by higher free immunoglobulin light chain levels (r = 0.509), but not by altered expression of Ki-67 (r= 0.033; Figure S1A+B). Together, these data underline the notion of ^11^C-MET being a promising marker for myeloma-imaging.

**Table 2:** Patient characteristics.

**Patient no.**	**age**	**sex**	**diagnosis**	**Ig**	**DS stage**	**initial diagnosis**	**cytogenetic alterations**
**1**	69	♀	MM	κ light chains	IIIB	06/2012	del13q; t(4;14)
**2**	61	♂	MGUS	n.d.	n.d.	2012	n.d.
**3**	73	♀	MGUS	IgG κ	n.d.	n.d.	n.d.
**4**	70	♀	MM	IgA λ	II A	01/2011	n.d.
**5**	80	♂	MM	IgG κ	I	07/2012	n.d.
**6**	41	♂	MM	IgG κ	IIA	12/2011	hyperdiploid
**7**	55	♂	MM	IgG κ	n.d.	08/2012	normal
**9**	71	♀	MM	IgG κ	III A	12/2011	del13q
**10**	62	♂	MM	IgA λ	III A	n.d.	hyperdiploid
**11**	64	♂	MM	IgG κ	III A	08/2012	del13q
**12**	62	♂	MM	IgG κ	IIIA	10/2012	normal
**13**	76	♂	MM	IgG λ	III A	10/2003	normal
**14**	64	♂	MM	IgA κ	IA	12/2002	del13q
**15**	73	♂	MM	IgG κ	IIIA	07/2006	del13q; t(11;14)
**16**	77	♂	MM	λ light chains	n.d.	06/2008	n.d.
**17**	65	♀	MM	IgG λ	IIIB	02/2009	normal
**18**	66	♂	MM	IgG κ	IIA	07/2006	n.d.
**19**	78	♂	MM	IgG κ	IIA	2006	n.d.
**20**	66	♀	MM	IgG λ	IIIA	1997	del13q14; t(4;14)
**21**	72	♂	MM	IgG κ	IIIA	04/1999	n.d.
**22**	53	♂	MM	IgA λ	IIIB	06/2007	n.d.
**23**	57	♀	MM	IgG κ	IA	06/2010	del13q14; t(11;14)
**24**	59	♂	MM	IgG λ	IIIA	04/2013	t(11;14);t(14q32) tri13q14
**25**	73	♀	MM	IgA κ	IIIA	07/2013	n.d.
**26**	54	♀	MM	IgG λ	II	12/2008	n.d.

## Discussion

Despite limited sensitivity and specificity, whole body x-ray is still considered as standard imaging test for detecting bone disease. The role of functional imaging in this scenario has not been clearly defined yet [6,16]. There is a growing body of evidence though that molecular imaging techniques, such as dynamic contrast-enhanced magnetic resonance imaging (MRI) or PET/computed tomography (PET/CT), might prove beneficial for discriminating active lesions from indolent ones, for assessment of treatment response and for therapeutic management of MM [7,8,10,17-22]. ^18^F-FDG-PET/CT has even been described as an emerging modality for imaging patients with multiple myeloma by the International Myeloma Working Group (IMWG). However, the concept of increased glucose metabolism as a surrogate for myeloma viability is hampered by non-specific retention of ^18^F-FDG in inflammatory lesions and reduced sensitivity in diffuse bone marrow infiltration. Moreover, several functional imaging approaches might be needed to accurately reflect tumor heterogeneity in MM [6,11,18].

In this study assessing the utility of alternative, potentially more specific imaging biomarkers for PET imaging, we have demonstrated a significantly higher retention of the radiolabeled amino acid ^11^C-MET in biologically diverse myeloma cells. In established cell lines, uptake of ^11^C-MET exceeded maximal ^18^F-FDG retention already after short incubation time and reached an approximately 1.5- to 5-fold higher uptake as compared to ^18^F-FDG and other tracers studied. Our data suggest that PET using ^11^C-MET as surrogate marker for paraprotein biosynthesis and amino acid turnover may outperform the current practice of imaging MM glucose use. These findings were recapitulated in primary MM cells derived from patients, providing further evidence of the utility of the proposed approach for MM imaging. 

Imaging paraprotein biosynthesis as read-out for viable myeloma lesions is supported by two recently published pilot clinical trials reporting an equal or even greater number of lesions in patients with plasma cell malignancies detected by ^11^C-MET-PET, as compared to ^18^F-FDG-PET [23,24]. Together, these encouraging results warrant larger prospective clinical trials to corroborate the initial findings and to further investigate the clinical value of ^11^C-MET-PET in non- or oligo-secretory myelomas as well as in the setting of dedifferentiated extramedullary disease. Furthermore, due to higher retention in myeloma cells, ^11^C-MET might prove useful for the detection of diffuse bone marrow involvement, a setting which is known as a weakness of ^18^F-FDG-PET imaging [16].

Importantly, in our study two distinct groups of cell lines could be discriminated on basis of ^11^C-MET retention: enhanced ^11^C-MET uptake tended to match with higher levels of intracellular immunoglobulin light chains, higher CD138 and CXCR4 expression on the cell surface and presence of cytogenetic aberrations associated with worse prognosis (t(4;14) in OPM-2). As immunoglobulin synthesis is a hallmark of MM, increased ^11^C-MET retention might thus be explained by at least partial incorporation into (para-) proteins, as has been shown for other tumor entities [25,26]. Molecules mediating the interaction between myeloma cells and bone marrow stromal cells, immunoglobulin levels and cytogenetic alterations are important determinants of myeloma pathology and serve as markers for disease activity and/or aggressiveness [27-31]. Based on this, the potential association of CD138, CXCR4 and intracellular immunoglobulins with ^11^C-MET uptake we found here, might allow for non-invasive risk stratification of the individual patient and response monitoring using imaging with PET/CT. Our data further suggest that relative ^11^C-MET uptake might be able to reflect myeloma tumor biology and, hence, might facilitate assessment of myeloma heterogeneity and discrimination of tumor subtypes.

The precise role of CD138 and CXCR4 in myeloma pathology and management remains to be determined though. With the introduction of very specific, targeted radiotracers, such as radiolabeled antibodies or artificial ligands (e.g. CXCR4 antagonists [32,33] or anti-CD138 antibodies [34,35]), these two factors present interesting targets for further research and potential theranostic applications [35-39]. As CXCR4 expression regulates myeloma cell homing and has very recently been linked to MM prognosis [40], this marker might further be useful for discriminating intra- and extramedullary MM lesions [41]. 

Although our data suggest that more aggressive cells with a high uptake of ^11^C-Methionine feature a higher proliferation rate and higher levels of intracellular immunoglobulin light chains (OPM-2), the alternate hypothesis, that a reduction of immunoglobulin production is accompanied by enhanced proliferation in more aggressive myelomas, is plausible as well. Accordingly, we found a partial connection of immunoglobulin levels and ^11^C-MET uptake in patient-derived primary cells, but there was no statistically significant correlation. When comparing patients diagnosed with MGUS (patients no. 2, 3) to patients with aggressive symptomatic myeloma (translocation t(4;14); patients no. 1, 20), degree of bone marrow infiltration and Ki-67 index are lower in MGUS, but none of the other parameters described distinguishes between the asymptomatic precursor form and full-blown myeloma (table S1). Based on the data shown here this conflict cannot be unequivocally answered, particularly due to the limited sample size of our study. It also has to be considered that multiple myeloma is a very heterogenous disease. Attempts to stratify myeloma patients into risk groups have hardly been successful so far. Therefore it is conceivable that there simply is no general pattern characterizing a certain type of myeloma, but many different individual presentations in a longitudinal follow-up, underlining the need for individualized patient management.

It can be speculated that the minimal cell uptake of ^18^F-FET, as observed in our study, is due to its less efficient transport into cells caused by the ^18^F-linker. Furthermore, myeloma cells predominantly express the large amino acid transporter 1 (LAT1) and tyrosine preferentially enters cells *via* LAT2 [42]. Although the underlying pathophysiological mechanism remains unclear, ^18^F-FET does not seem to be a promising candidate biomarker in myeloma imaging. 

In conclusion, ^11^C-MET might be superior to ^18^F-FDG regarding detection of active myeloma lesions. The higher sensitivity of ^11^C-MET could prove useful to overcome limitations of standard ^18^F-FDG-PET/CT including detection of minimal bone marrow infiltration, diffusely disseminated intramedullary disease and/or detection of myeloma cells with just marginally increased metabolism. The possibility of a connection between ^11^C-MET uptake and intracellular immunoglobulin light chain, CD138 and CXCR4 levels raises potential for patient risk stratification, response monitoring and treatment individualization. 

## Supporting Information

Figure S1 **Free immunoglobulin light chain and Ki-67 expression in selected CD138^+^-plasma cell samples as a function of ^11^C-MET uptake.** Levels of free immunoglobulin light chains in serum and percentage of Ki-67^+^ cells in bone marrow biopsies were obtained from routine diagnostic workup of selected patients (patients no. 13, 16, 17, 18, 19, 21, 22, 26). Correlation analysis according to Pearson of free immunoglobulin light chains (r = 0.509; A) or Ki-67 expression (r = 0.033; B) with ^11^C-MET uptake and of free immunoglobulin light chains and Ki-67 (r = 0.124; C) in CD138^+^-plasma cell samples is shown.
(DOCX)

Table S1 **Clinical presentation of MGUS vs. MM.**
(DOCX)
