# Supplementary material for: Targeting Paraprotein Biosynthesis for Non-Invasive Characterization of Myeloma Biology
Source: PLoS One. 2013 Dec 23;8(12):e84840. doi: 10.1371/journal.pone.0084840 (PMC3871597; doi:10.1371/journal.pone.0084840)
Supplement: Figure S1 — Free immunoglobulin light chain and Ki-67 expression in selected CD138+-plasma cell samples as a function of 11C-MET uptake. Levels of free immunoglobulin light chains in serum and percentage of Ki-67+ cells in bone marrow biopsies were obtained from routine diagnostic workup of selected patients (patients no. 13, 16, 17, 18, 19, 21, 22, 26). Correlation analysis according to Pearson of free immunoglobulin light chains (r = 0.509; A) or Ki-67 expression (r = 0.033; B) with 11C-MET uptake and of free immunoglobulin light chains and Ki-67 (r = 0.124; C) in CD138+-plasma cell samples is shown. (DOCX) [file pone.0084840.s001.docx]

**
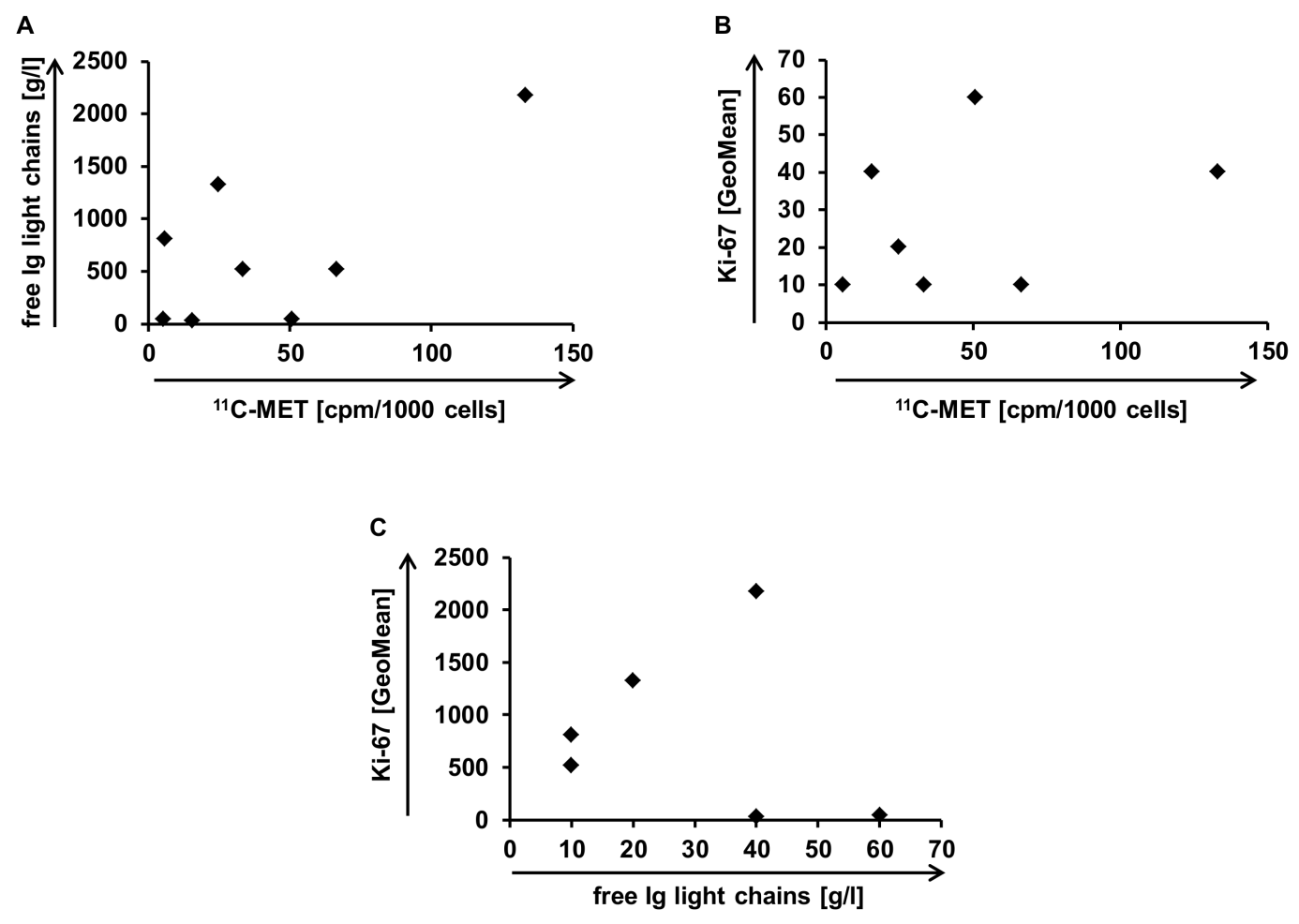
**

**Supplementary Figure S1. Free immunoglobulin light chain and Ki-67 expression in selected CD138^+^-plasma cell samples as a function of ^11^C-MET uptake**
