# Supplementary material for: Targeting Paraprotein Biosynthesis for Non-Invasive Characterization of Myeloma Biology
Source: PLoS One. 2013 Dec 23;8(12):e84840. doi: 10.1371/journal.pone.0084840 (PMC3871597; doi:10.1371/journal.pone.0084840)
Supplement: Table S1 — Clinical presentation of MGUS vs. MM. (DOCX) [file pone.0084840.s002.docx]

**Supplementary Table 1. Clinical presentation of MGUS *vs*. MM**

| **patient no.** | **#2** | **#3** | **#1** | **#20** |
| --- | --- | --- | --- | --- |
| **diagnosis** | **MGUS** | **MGUS** | **MM** | **MM** |
| **Ig** | λ-light chain | IgG κ | λ-light chain | IgG λ |
| **cytogenetics** | n.d. | n.d. | del13q; t(4;14) | del13q; t(4;14) |
| **BM Infiltration** | 15% | 5% | 60% | 100% |
| **Ki-67** | 10% | 10% | 20% | 20% |
| **M-Gradient** | n.d. | n.d. | n.d. | n.d. |
| **free κ light chains [mg/l]** | 0.73 | 9.09 | 1.07 | 6.66 |
| **free λ light chains [mg/l]** | 693.49 | 1.73 | 2453.00 | 368.80 |
| **κ/λ-ratio** | 0 | 5.25 | 0 | 0.02 |
| **^11^C-MET [cpm/1000cells]** | n.d. | n.d. | n.d. | n.d. |
| **^18^F-FDG [cpm/1000cells]** | 29.95 | 88.13 | 103.34 | 27.93 |
